# Changes in pulmonary function and their correlation with dose–volume parameters in patients undergoing stereotactic body radiotherapy for lung cancer

**DOI:** 10.1093/jrr/rraa131

**Published:** 2021-01-22

**Authors:** Shinya Takemoto, Yuta Shibamoto, Chisa Hashizume, Akifumi Miyakawa, Taro Murai, Takeshi Yanagi, Chikao Sugie, Aiko Nagai

**Affiliations:** Department of Radiology, Nagoya City University Graduate School of Medical Sciences, 1 Kawasumi, Mizuho-cho, Mizuho-ku, Nagoya, Aichi, 467-8601, Japan; Department of Radiology, Fujieda Heisei Memorial Hospital, 123-1, Mizukami, Fujieda, Shizuoka, 426-8662, Japan; Department of Radiology, Nagoya City University Graduate School of Medical Sciences, 1 Kawasumi, Mizuho-cho, Mizuho-ku, Nagoya, Aichi, 467-8601, Japan; Nagoya Radiosurgery Center, Nagoya Kyoritsu Hospital, 1-172, Hokke, Nakagawa-ku, Nagoya, Aichi, 454-0933, Japan; Department of Radiation Oncology, National Hospital Organization Nagoya Medical Center, 4-1-1, Sannomaru, Naka-ku, Nagoya, Aichi, 460-0001, Japan; Department of Radiology, Nagoya City University Graduate School of Medical Sciences, 1 Kawasumi, Mizuho-cho, Mizuho-ku, Nagoya, Aichi, 467-8601, Japan; Narita Memorial Proton Center, 78 Shirakawa-cho, Toyohashi, Aichi, 441-8021, Japan; Department of Radiology, Nagoya Daini Red Cross Hospital, 2-9 Myoken-cho, Showa-ku, Nagoya, Aichi, 466-8650, Japan; Department of Radiation Oncology, Nishichita General Hospital, 3-1-1 Nakanoike, Tokai, Aichi, 477-8522, Japan

**Keywords:** lung cancer, stereotactic radiation therapy, pulmonary function test, dose–volume parameters

## Abstract

It is desirable to estimate the degree of the decrease in pulmonary function before lung stereotactic body radiation therapy (SBRT) especially for patients with poor pulmonary function. The purpose of this study was to investigate whether decreases in pulmonary function after SBRT may be predicted from radiation dose–volume parameters.

A total of 70 patients undergoing SBRT were evaluated for changes in pulmonary function. Of these, 67 had primary lung cancer and 3 had lung metastasis. Twenty-six (37%) patients had chronic obstructive pulmonary disease. Pulmonary function tests (PFTs) were performed shortly before and at 18–24 months after SBRT. Radiation pneumonitis was Grade 2 in 10 patients and Grade 3 in 1. Mean forced vital capacity (FVC) decreased from 2.67 to 2.51 L (*P* < 0.01) and mean forced expiratory volume in 1 s (FEV1) decreased from 1.80 to 1.72 L (*P* < 0.01). Planning target volume (PTV) was correlated with changes in FVC. Changes in percent predicted FVC were correlated with %V_5Gy_ (% of lung volume receiving > 5 Gy) and %V_40Gy_. Although the correlation was not significant, the %V_20Gy_ value was the closest to the percent reduction in predicted FVC; %V_20Gy_ of 10% tended to be associated with ~10% reduction in predicted FVC. Patients with poor pulmonary function did not necessarily show greater decreases in each PFT parameter. Decreases in FVC and FEV1 were within previously reported ranges. PTV was associated with decreases in FVC. The %V_20Gy_ value was closest to the percentage decrease in predicted FVC.

## INTRODUCTION

Stereotactic body radiation therapy (SBRT) is almost established for the treatment of early-stage primary and metastatic lung cancer. The reported results of SBRT suggest outcomes nearly equal to those of surgery [1–6], but complications are somewhat different between the two modalities. The major adverse effects of SBRT are radiation fibrosis following radiation pneumonitis and rib fractures. Since the volume of the lung is considered to decrease due to radiation fibrosis, it may be desirable to estimate the degree of the decrease in pulmonary function before SBRT to provide patients with this information. This is especially important when treating patients who already have reduced pulmonary function. We therefore examined the association between the decrease in parameters of the pulmonary function test (PFT), such as forced vital capacity (FVC) and forced expiratory volume in 1 s (FEV1), and dose–volume histogram (DVH) parameters for the lung such as V_40Gy_ (percentage of lung volume receiving ≥40 Gy).

Several groups have investigated the changes in pulmonary function before and after SBRT; most of their results indicate slight to moderate decreases in some PFT parameters [7–9]. To the best of our knowledge, however, only a few groups have attempted to correlate the degree of decrease in PFT parameters with DVH parameters [8, 10], and none have attempted to correlate changes in pulmonary function with DVH parameters in individual patients. Therefore, the present study aimed to evaluate PFT results before and after SBRT and to determine the correlations, if any, between the PFT results and DVH data.

## MATERIALS AND METHODS

### Study design and eligibility criteria

This was a prospective study approved by the institutional review board (approval number: 60-18-0150) and was conducted at two institutions. Written informed consent was obtained from all patients. Nearly all patients had entered our prospective studies of SBRT for stage I non-small-cell lung cancer (NSCLC) or solitary lung metastasis [6, 11, 12].

Eligibility criteria of the present study were: (i) treatment performed as per our protocols [6, 11, 12]; (ii) only one pulmonary lesion treated; (iii) no history of lung surgery or radiotherapy before and after SBRT until the post-SBRT PFT; (iv) no local, regional or distant relapse before post-SBRT PFT; and (v) informed consent. Exclusion criteria were: (i) doubtful reliability of PFT results due to advanced age or cognitive function; and (ii) improvement of pulmonary conditions such as atelectasis by SBRT. Taking the results of previous studies [7–10] into account, mean FVC and FEV1 reductions of 5% were assumed, and to statistically validate the reductions at least 63 patients were considered necessary, assuming a standard deviation (SD) of 10%, with an alpha error of 5% and a beta error of 20% [13]. After confirming the decreases in the PFT data and correlations between the PFT results and some DVH parameters, the study aimed to determine DVH parameters showing the percentage value closest to the percentage decrease in PFT data.

Pre-SBRT PFT was mandatory in our studies of SBRT. Patients who had undergone SBRT for primary or metastatic lung cancer and agreed to enter this study were evaluated for changes in pulmonary function. Sialylated carbohydrate antigen KL-6 (KL-6) was measured before SBRT. The tumor location was classified into central or peripheral in accordance with the Radiation Therapy Oncology Group criteria [14].

### Treatment

Our SBRT methods were described in detail previously [6, 11, 12]. Briefly, the visible gross tumor volume on CT during three phases (under normal breathing, and with breath-holding during the expiratory and inspiratory phases) was superimposed to represent the internal target volume (ITV). The planning target volume (PTV) margin for the ITV was 5 mm in the lateral and anteroposterior directions and 5–10 mm in the craniocaudal direction. The dose calculation algorithm was pencil beam convolution with Batho power law correction before November 2008, and anisotropic analytical algorithm thereafter.

SBRT was delivered in four fractions with three coplanar and four non-coplanar static 6-MV beams. The total dose at the isocenter was 44 Gy (treated before November 2008) or 48 Gy (after November 2008) for tumors with a maximum diameter <1.5 cm, and 48 or 50 Gy for tumors of 1.5–3 cm. For those >3 cm, the total dose was 52 Gy. Thus, biological equivalent doses differed depending on the prescribed doses. The inter-fraction interval was set to ≥72 h based on radiobiological considerations [15]. After SBRT, chest and upper abdominal CT was performed at 2-month intervals until 6 months, and every 2–4 months thereafter. Radiation pneumonitis was evaluated by these images and graded according to the Common Toxicity Criteria for Adverse Events version 4.0.

### PFT

PFTs were performed just before SBRT and between 18 and 24 months (median, 20 months) after SBRT. This timing was chosen because radiation fibrosis changes seemed to be complete by 18 months. The PFT equipment model was a Chestac-8800/8900, (Chest, Tokyo, Japan) or SP-350COPD/370COPD (Fukuda Denshi, Tokyo, Japan); the model of the same manufacturer was used for each patient. In this study, we evaluated FVC, FEV1 and FEV% in 1 s (FEV1%). Predicted values of FVC, FEV1 and FEV1% were calculated using the LMS method for Japanese adults [16].

### Statistical analysis

Changes in PFT between pre- and post-SBRT examinations were evaluated using the paired t-test. Correlations between patient characteristics and PFT changes were examined by the Mann–Whitney U-test and Spearman’s rank correlation. All statistical analyses were two-sided and performed with EZR (Saitama Medical Center, Jichi Medical University, Saitama, Japan), which is a graphical user interface for R (The R Foundation for Statistical Computing, Vienna, Austria). More precisely, it is a modified version of R commander designed to add statistical functions frequently used in biostatistics [17]. A *P* value < 0.05 was considered significant.

## RESULTS

Between August 2010 and November 2016, 75 patients agreed to enter this study. Five of them were excluded because their PFT results seemed unreliable, with >30% increase in FVC at the second PFT. Ultimately, 70 patients were evaluated in the present study (Table 1). The median age of all patients was 78 years. A total of 67 patients had primary lung cancer and 3 had lung metastasis. The patients with primary lung cancer were clinically staged as T1a (*n* = 24), T1b (*n* = 27) or T2a (*n* = 16) according to the UICC cancer staging classification, 7th edition. In all, 54 patients had histologically-proven lung cancer (adenocarcinoma in 37, squamous cell carcinoma in 13, large cell carcinoma in 2, small cell carcinoma in 1 and basaloid carcinoma in 1). Twenty-six (37%) patients had chronic obstructive pulmonary disease (COPD). Radiation pneumonitis was Grade 0 or 1 in 59 patients, Grade 2 in 10 and Grade 3 in 1. Two patients had mild interstitial shadows with normal KL-6 levels, but they did not develop Grade ≥ 2 radiation pneumonitis. The patient developing Grade 3 pneumonitis required home oxygen therapy after development of pneumonitis. Grade ≥ 2 radiation pneumonitis occurred in 5 of 48 patients with an upper lobe tumor and 6 of 22 patients with a middle or lower lobe tumor (*P* = 0.09), in 7 of 61 patients with a peripheral tumor and in 4 of 9 patients with a central tumor (*P* = 0.03), and in 4 of 26 patients with COPD and 7 of 44 patients without COPD (*P* = 1.0).

**Table 1:** Patient characteristics

**Characteristics**	**Number**
Total patients	70
Age years range (median)	56–89 (78)
Sex male/female	44/26
Primary/metastatic	67/3
Clinical T stage, T1a/T1b/T2a	24/27/16
Histology	
Adenocarcinoma	38
Squamous cell carcinoma	15
Others	4
Undifferentiated	13
Involved robe upper/middle or lower	48/22
Tumor location periphery/center	61/9
Smoking history yes/no	43/22
Brinkman index ≥/< 400	37/28
COPD yes/no	26/44
Interstitial pneumonia yes/no	5/65
KL-6 (U/ml) range (median)	132–1527 (286)
Prescribed dose (4 fractions)	
44/48/50/52 Gy	1/19/38/12
PTV (cm^3^) range (median)	8.7–106.5 (41.9)
Total lung volume (cm^3^) range (median)	1297–5064 (2744)
Lung %V_5Gy_ (%) range (median)	8.1–40.1 (18.5)
Lung %V_15Gy_ (%) range (median)	3.0–19.7 (7.6)
Lung %V_20Gy_ (%) range (median)	2.3–16.1 (5.4)
Lung %V_30Gy_ (%) range (median)	1.3–10.4 (3.2)
Lung %V_40Gy_ (%) range (median)	0.7–6.7 (2.2)

Pre- and post-SBRT PFT data are shown in Table 2. When pre- and post-SBRT data were compared, the mean FVC decreased from 2.67 to 2.51 L (*P* < 0.01) and the mean FEV1 decreased from 1.80 to 1.72 L (*P* < 0.01). There was no significant change in mean FEV1% (*P* = 0.21). Percent predicted FVC and FEV1 also showed significant reductions (*P* < 0.01 and *P* = 0.03, respectively). Mean change rates in individual patients ([post − baseline]/baseline; %) were −5.9% for FVC, −4.6% for FEV1 and +2.1% for FEV1%. Mean changes in the percent predicted values of individual patients were −4.9% for FVC, −3.3% for FEV1 and +2.4% for FEV1%.

**Table 2:** Results of pulmonary function tests for all patients

	Before SBRT	After SBRT	*P* ^a^	Individual change^b^
	Mean (SD) range			Mean (SD) range
FVC	2.67 (0.73) L 1.23–4.48	2.51 (0.74) L 0.94–4.55	<0.01	−5.9 (11.1) % −33.3 to +17.1
FEV1	1.80 (0.64) L 0.65–3.47	1.72 (0.65) L 0.59–3.60	<0.01	−4.6 (12.1) % −45.5 to +39.4
FEV1%	67.0 (13.7) % 26.1–92.5	68.1 (14.7)% 22.9–97.4	0.21	+2.1 (13.1)% −41.7 to +45.2
FVC% predicted	92.0 (19.5) % 37.1–142.0	87.5 (20.3)% 25.1–146.2	<0.01	−4.9 (11.2)% −32.3 to +18.6
FEV1% predicted	80.9 (24.2) % 25.4–136.6	78.3 (24.9)% 20.7–134.3	0.03	−3.3 (12.3)% −44.4 to +41.9
FEV1% % predicted	87.4 (17.7)% 33.5–121.6	89.2 (19.3)% 30.1–127.3	0.12	+2.4 (13.1)% −41.4 to +45.7

^a^Paired-t test.

^b^Individual change rates were calculated with [post − baseline]/baseline (%).

**Fig. 1.**
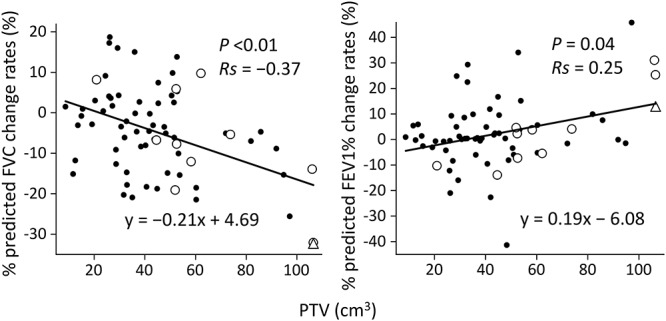
Scatter plot of PTV vs changes in percent predicted FVC and PTV vs changes in percent predicted FEV1%. Circles represent patients with Grade 2 radiation pneumonitis and triangles represent a patient with Grade 3 radiation pneumonitis. The solid lines are regression lines. *Rs* = Spearman’s rank correlation coefficient.

**Fig. 2.**
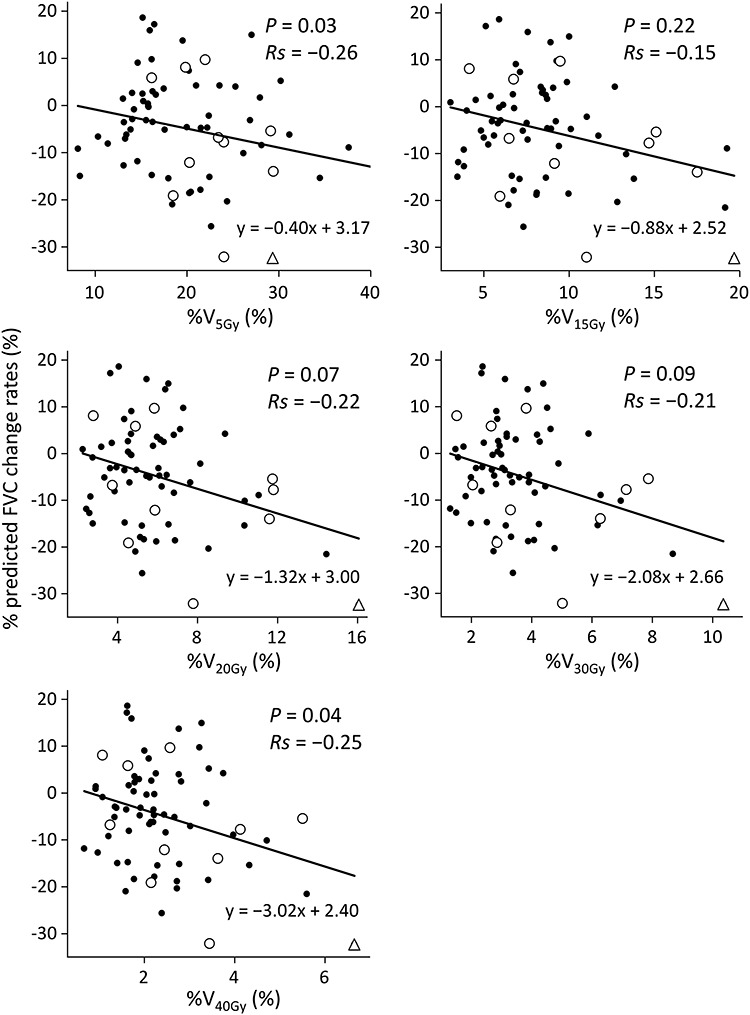
Scatter plot of changes in percent predicted FVC vs %V_5–40Gy_. Circles represent patients with Grade 2 radiation pneumonitis and triangles represent a patient with Grade 3 radiation pneumonitis. The solid lines are regression lines. *Rs* = Spearman’s rank correlation coefficient.

Associations between the change rates in predicted PFT data (%) and parameters including patient characteristics and dose distributions are shown in Table 3. FVC and FEV1 showed significant reductions in patients with a tumor located in the middle or lower lobe (*P* = 0.04 and 0.02, respectively). No correlation was found between PFT changes and presence of COPD, Brinkman Index or baseline KL-6 levels. Patients with poor pulmonary function did not necessarily show greater decrease in PFT; 20 patients whose pre-SBRT percent predicted FVC was <80% and 38 patients whose pre-SBRT percent predicted FEV1% was <70% showed no decrease in PFT parameters. No significant differences in PFT change rates were observed between patients with a peripheral tumor and those with a central tumor, although the latter patients tended to have a decreased FEV1 (*P* = 0.06). PTV was correlated with decreases in predicted FVC (*P* < 0.01) and increases in predicted FEV1% (*P* = 0.04) (Fig. 1). In the 11 patients with Grade ≥ 2 radiation pneumonitis, the lowest PTV was 21.0 cm^3^ and the second lowest was 44.6 cm^3^. Decreases in predicted FVC were correlated with %V_5Gy_ (% of lung volume receiving >5 Gy) and %V_40Gy_ (*P* = 0.03 and 0.04, respectively). Although the correlation was not significant (*P* = 0.07, Spearman’s rank correlation coefficient = −0.22), the %V_20Gy_ value on the regression line was the closest to the percent reduction in predicted FVC (Fig. 2); patients with %V20Gy of 10% tended to have ~10% reduction in predicted FVC after SBRT. Among the 11 patients with Grade ≥ 2 radiation pneumonitis, the lowest %V_5Gy_, %V_15Gy_, %V_20Gy_, %V_30Gy_ and %V_40Gy_ were 16.2, 4.2, 2.8, 1.5 and 1.1%, respectively.

**Table 3:** Correlation between patient characteristics and change rates of % predicted PFT data

Characteristics	*P* value (correlation coefficient^a^)		
	% Predicted FVC	% Predicted FEV1	% Predicted FEV1%
Age^a^	0.89 (0.02)	0.27 (0.13)	0.13 (0.18)
Sex^b^	0.31	0.93	0.24
Lobe upper vs middle/lower^b^	**0.04**	**0.02**	0.79
Location periphery vs center^b^	0.17	0.06	0.67
COPD yes vs no^b^	0.63	0.69	0.58
Brinkman index ≥ vs < 400^b^	0.33	0.76	0.18
KL-6 ≥ vs < 500^b^	0.17	0.42	0.87
PTV^a^	**<0.01** (−0.37)	0.30 (−0.13)	**0.04** (0.25)
Total lung volume^a^	0.60 (−0.06)	0.69 (0.05)	0.93 (0.01)
Lung %V_5Gy_^a^	**0.03** (−0.26)	0.15 (−0.18)	0.48 (0.09)
Lung %V_15Gy_^a^	0.22 (−0.15)	0.60 (−0.06)	0.16 (0.17)
Lung %V_20Gy_^a^	0.07 (−0.22)	0.31 (−0.12)	0.18 (0.16)
Lung %V_30Gy_^a^	0.09 (−0.21)	0.58 (−0.07)	0.08 (0.21)
Lung %V_40Gy_^a^	**0.04** (−0.25)	0.28 (−0.13)	0.19 (0.16)

^a^Spearman’s rank correlation.

^b^Mann–Whitney U test.

Bold text indicates a statistically significant difference with a *P* value less than 0.05.

## DISCUSSION

Clinically symptomatic pneumonitis has been reported to develop in <10% of patients after radiation therapy to the lung [18], but most patients develop pulmonary fibrosis as a late toxicity that can adversely affect lung function [19]. It is important to evaluate changes in lung function to provide appropriate care or treatment, since many patients undergoing SBRT already have poor lung function. Indeed, 29% of our patients had <80% of percent predicted FVC and 54% had <70% of percent predicted FEV1% before SBRT in this study.

Regarding previous studies on pulmonary function after lung SBRT, there was a significant decrease in FVC and FEV1 at 24 months after SBRT in one study [7]; percent predicted FVC and FEV1 also decreased but the difference was not significant. There appeared to be no significant PFT changes within a relatively short period (e.g. 1 year) [7, 10]. In another study, FEV1 tended to decrease, but not significantly, with a median time to most recent PFTs of 10.4 months [8]. In contrast, the RTOG 0236 study [20] showed no changes in FVC, FEV1 or FEV1% at 2 years after SBRT, which may have been due to the small number of patients. Takeda *et al*. [9] reported relatively long-term results with a median of 21.0 months after SBRT; they found significant decreases in FVC and FEV1, but these were not significant in COPD patients. However, the effects of aging on pulmonary function may also be involved in the decrease. In this study, the mean change rates in the predicted values in individual patients were −4.9% for FVC and −3.3% for FEV1, whereas their actual change rates were −5.9 and −4.6%, respectively. These data were close to those estimated when calculating the sample size, and we think that ~5% decreases in FVC and FEV1 are within acceptable ranges. Summarizing all these studies, SBRT may cause a slight decrease in pulmonary function, but the changes may be within an acceptable range. Patients with poor baseline PFT did not necessarily show a greater decrease in PFT parameters. This result may support the result from RTOG 0236 [20] that poor baseline PFT did not predict decreased overall survival after SBRT. Other studies also reported similar results regarding the safety of SBRT in patients with poor lung function [7, 8, 21].

Various factors have been reported to correlate with the severity of radiation pneumonitis, but conflicting data exist. Patients with a central tumor were reported to have a higher incidence of grade 3–5 toxicity [14], whereas this was not observed in another study [22]. In our study, FEV1 tended to decrease in patients with a central tumor (*P* = 0.06). COPD was associated with radiation pneumonitis in one study [23], whereas radiation pneumonitis after SBRT was relatively mild in patients with severe COPD in another study [24]. The latter may partly be attributed to the lower radiation doses used for COPD patients. In the present study, PFT results did not differ significantly between COPD and non-COPD patients. Pretreatment serum KL-6 levels were associated with symptomatic radiation pneumonitis in one study [25], but there was no difference in changes in PFT results due to KL-6 in our study.

V_20Gy_ and mean lung dose (MLD) have been reported to be predictors of radiation pneumonitis [26–28]. Regarding the correlation between PFT changes and DVH parameters of SBRT, Guckenberger *et al*. [29] reported that tumor size, PTV dose, MLD, and absolute and relative V_5–70Gy_ were not correlated with decreased FEV1%. Ohashi *et al*. [10] found no correlation between PFT parameters (total lung capacity, vital capacity and FEV1) and PTV, V_15–30Gy_ or MLD. In contrast, one study showed a correlation between decrease in FEV1% and parameters of V_5Gy_ and V_10Gy_ [8]. In the present study, a negative correlation was observed between PTV and FVC changes (*P* < 0.01), and a positive correlation was found between PTV and FEV1% changes (*P* = 0.04). However, since 31% (22/70) of the patients had an increase in FVC after SBRT, these results must be interpreted cautiously. Further studies are necessary to validate this observation. Nevertheless, the decrease in predicted FVC was correlated with %V_5Gy_ and %V_40Gy_. Although the correlation was not significant for %V_15Gy_, %V_20Gy_ or %V_30Gy_, the %V_20Gy_ value on the regression line was the closest to the percent reduction in predicted FVC (Fig. 2). However, because of the large variations in PTF data among patients, this may not be applicable to individual patients. Nevertheless, our results may be of some help in predicting PFT changes before SBRT and in explaining the complications of SBRT to patients.

This study had some limitations. PFT was performed only once in each pre- and post-SBRT examination. Therefore, the patient conditions at examination and unfamiliarity of patients with PFT may have produced large variations in the PFT data. When conducting such a study in the future, we feel that PFT should be conducted at least twice on the day of examination. Furthermore, we investigated only FVC, FEV1 and FEV1%, and did not include other parameters such as diffusing capacity of the lung for carbon monoxide (DLCO), which may show a more detailed correlation. Some studies reported a decrease in DLCO [20, 21, 30], which may be partly explained by patients’ histories of cardiopulmonary disease. In addition, correlation between MLD and PFT results was not investigated. In analysis of the data at one of the two participating institutions (*n* = 36), MLD was correlated with percent predicted FVC (*P* = 0.013, correlation coefficient = −0.41) but not with percent predicted FEV1 (*P* = 0.28) and percent predicted FEV1% (*P* = 0.10). In future studies investigating the correlation between DVH parameters and PFT results, it might be desirable to include MLD.

## CONCLUSION

FVC and FEV1 decreased significantly after SBRT for lung cancer, but these changes appeared to be within previously reported ranges. PTV was associated with decreased FVC. Although it seemed difficult to predict the degree of decrease in pulmonary function from DVH data in individual patients, the %V_20Gy_ value was closest to the percent decrease in predicted FVC.

## References

[1] Onishi H, Shioyama Y, Matsumoto Y et al. Stereotactic body radiotherapy in patients with lung tumors composed of mainly ground-glass opacity. J Radiat Res 2020;61:426–30. 32219316 10.1093/jrr/rraa015 PMC7299254

[2] Matsuo Y, Chen F, Hamaji M et al. Comparison of long-term survival outcomes between stereotactic body radiotherapy and sublobar resection for stage I non-small-cell lung cancer in patients at high risk for lobectomy: A propensity score matching analysis. Eur J Cancer 2014;50:2932–8. 25281527 10.1016/j.ejca.2014.09.006

[3] Chang JY, Senan S, Paul MA et al. Stereotactic ablative radiotherapy versus lobectomy for operable stage I non-small-cell lung cancer: A pooled analysis of two randomised trials. Lancet Oncol 2015;16:630–7. 25981812 10.1016/S1470-2045(15)70168-3 PMC4489408

[4] Nagata Y, Hiraoka M, Shibata T et al. Prospective trial of stereotactic body radiation therapy for both operable and inoperable T1N0M0 non-small cell lung cancer: Japan clinical oncology group study JCOG0403. Int J Radiat Oncol Biol Phys 2015;93:989–96. 26581137 10.1016/j.ijrobp.2015.07.2278

[5] Shibamoto Y, Hashizume C, Baba F et al. Stereotactic body radiotherapy using a radiobiology-based regimen for stage I non-small cell lung cancer: Five-year mature results. J Thorac Oncol 2015;10:960–4. 26001145 10.1097/JTO.0000000000000525

[6] Miyakawa A, Shibamoto Y, Baba F et al. Stereotactic body radiotherapy for stage I non-small-cell lung cancer using higher doses for larger tumors: Results of the second study. Radiat Oncol 2017;12:152. 28893300 10.1186/s13014-017-0888-7 PMC5594596

[7] Stone B, Mangona VS, Johnson MD et al. Changes in pulmonary function following image-guided stereotactic lung radiotherapy. Neither lower baseline nor post-SBRT pulmonary function are associated with worse overall survival. J Thorac Oncol 2015;10:1762–9. 26334751 10.1097/JTO.0000000000000670

[8] Stephans KL, Djemil T, Reddy CA et al. Comprehensive analysis of pulmonary function test (PFT) changes after stereotactic body radiotherapy (SBRT) for stage I lung cancer in medically inoperable patients. J Thorac Oncol 2009;4:838–44. 19487961 10.1097/JTO.0b013e3181a99ff6

[9] Takeda A, Enomoto T, Sanuki N et al. Reassessment of declines in pulmonary function ≥1 year after stereotactic body radiotherapy. Chest 2013;143:130–7. 22722234 10.1378/chest.12-0207

[10] Ohashi T, Takeda A, Shigematsu N et al. Differences in pulmonary function before vs. 1 year after hypofractionated stereotactic radiotherapy for small peripheral lung tumors. Int J Radiat Oncol Biol Phys 2005;62:1003–8. 15990001 10.1016/j.ijrobp.2004.12.050

[11] Baba F, Shibamoto Y, Tomita N et al. Stereotactic body radiotherapy for stage I lung cancer and small lung metastasis: Evaluation of an immobilization system for suppression of respiratory tumor movement and preliminary results. Radiat Oncol 2009;4:15. 19476628 10.1186/1748-717X-4-15 PMC2694202

[12] Shibamoto Y, Hashizume C, Baba F et al. Stereotactic body radiotherapy using a radiobiology-based regimen for stage I nonsmall cell lung cancer. A multicenter study. Cancer 2012;118:2078–84. 22009495 10.1002/cncr.26470

[13] University of California, San Francisco. Sample Size Calculators. http://www.sample-size.net/sample-size-means/ (15 Jan 2020, date last accessed).

[14] Timmermann R, McGarry R, Yiannoutsos C et al. Excessive toxicity when treating central tumors in a phase II study of stereotactic body radiation therapy for medically inoperable early-stage lung cancer. J Clin Oncol 2006;24:4833–9. 17050868 10.1200/JCO.2006.07.5937

[15] Shibamoto Y, Miyakawa A, Otsuka S et al. Radiobiology of hypofractionated stereotactic radiotherapy: What are the optimal fractionation schedules? J Radiat Res 2016;57:i76–82. 27006380 10.1093/jrr/rrw015 PMC4990108

[16] Kubota M, Kobayashi H, Quanjer PH et al. Reference values for spirometry, including vital capacity, in Japanese adults calculated with the LMS method and compared with previous values. Respir Investig 2014;52:242–50. 10.1016/j.resinv.2014.03.003 24998371

[17] Kanda Y. Investigation of the freely-available easy-to-use software “EZR” (easy R) for medical statistics. Bone Marrow Transplant 2013;48:452–8. 23208313 10.1038/bmt.2012.244 PMC3590441

[18] Marks LB, Bentzen SM, Deasy JO et al. Radiation dose-volume effects in the lung. Int J Radiat Oncol Biol Phys 2010;76:S70–6. 20171521 10.1016/j.ijrobp.2009.06.091 PMC3576042

[19] Huang K, Dahele M, Senan S et al. Radiographic changes after lung stereotactic ablative radiotherapy (SABR)--can we distinguish recurrence from fibrosis? A systematic review of the literature. Radiother Oncol 2012;102:335–42. 22305958 10.1016/j.radonc.2011.12.018

[20] Stanic S, Paulus R, Timmerman RD et al. No clinically significant changes in pulmonary function following stereotactic body radiation therapy for early- stage peripheral non-small cell lung cancer: An analysis of RTOG 0236. Int J Radiat Oncol Biol Phys 2014;88:1092–9. 24661663 10.1016/j.ijrobp.2013.12.050 PMC4058437

[21] Henderson M, McGarry R, Yiannoutsos C et al. Baseline pulmonary function as a predictor for survival and decline in pulmonary function over time in patients undergoing stereotactic body radiotherapy for the treatment of stage I non-small-cell lung cancer. Int J Radiat Oncol Biol Phys 2008;72:404–9. 18394819 10.1016/j.ijrobp.2007.12.051

[22] Chang JY, Liu H, Balter P et al. Clinical outcome and predictors of survival and pneumonitis after stereotactic ablative radiotherapy for stage I non-small cell lung cancer. Radiat Oncol 2012;7:152. 22963661 10.1186/1748-717X-7-152 PMC3444889

[23] Inoue T, Shiomi H, Oh RJ. Stereotactic body radiotherapy for stage I lung cancer with chronic obstructive pulmonary disease: Special reference to survival and radiation-induced pneumonitis. J Radiat Res 2015;56:727–34. 25887042 10.1093/jrr/rrv019 PMC4497392

[24] Takeda A, Kunieda E, Ohashi T et al. Severe COPD is correlated with mild radiation pneumonitis following stereotactic body radiotherapy. Chest 2012;141:858–66. 21885726 10.1378/chest.11-1193

[25] Iwata H, Shibamoto Y, Baba F et al. Correlation between the serum KL-6 level and the grade of radiation pneumonitis after stereotactic body radiotherapy for stage I lung cancer or small lung metastasis. Radiother Oncol 2011;101:267–70. 21640420 10.1016/j.radonc.2011.05.031

[26] Nakamura M, Nishimura H, Nakayama M et al. Dosimetric factors predicting radiation pneumonitis after CyberKnife stereotactic body radiotherapy for peripheral lung cancer. Br J Radiol 2016;89:20160560. 27805837 10.1259/bjr.20160560 PMC5604921

[27] Barriger RB, Forquer JA, Brabham JG et al. A dose-volume analysis of radiation pneumonitis in non-small cell lung cancer patients treated with stereotactic body radiation therapy. Int J Radiat Oncol Biol Phys 2012;82:457–62. 21035956 10.1016/j.ijrobp.2010.08.056

[28] Matsuo Y, Shibuya K, Nakamura M et al. Dose-volume metrics associated with radiation pneumonitis after stereotactic body radiation therapy for lung cancer. Int J Radiat Oncol Biol Phys 2012;83:e545–9. 22436782 10.1016/j.ijrobp.2012.01.018

[29] Guckenberger M, Klement RJ, Kestin LL et al. Lack of a dose-effect relationship for pulmonary function changes after stereotactic body radiation therapy for early-stage non-small cell lung cancer. Int J Radiat Oncol Biol Phys 2013;85:1074–81. 23154077 10.1016/j.ijrobp.2012.09.016

[30] Lischalk JW, Woo SM, Kataria S et al. Long-term outcomes of stereotactic body radiation therapy (SBRT) with fiducial tracking for inoperable stage I non-small cell lung cancer (NSCLC). J Radiat Oncol 2016;5:379–87. 28018523 10.1007/s13566-016-0273-4 PMC5149392

